# Inflammation

**Published:** 1864-02

**Authors:** W. H. Atkinson


### INFLAMMATION.

Read before the Society of Dental Surgeons of New York, on the evening of Jan. 27, 1864.
BY W. H. ATKINSON, M.D., D.D.S.

The inflammatory process may be fractional or complete, the former indicating arrest at some one of its stages before the final destruction of the part has been effected.

There can be no disease in the system, without the presence of some stage of this process. It, therefore, becomes of the highest possible moment to fully understand the institution, progress and regress of these stages, in combination, and alone, so far as cognizable by our perception.

Two prime conditions are necessary to the production and maintainance of this process, to-wit: perversion of the neural *and* vascular circulations. Just here a whole chapter of animadversion against existing pathological nomenclature might pertinently be indulged; but we will refrain therefrom, and give what we deem a better, because simpler and more definite nomenclature, that we may be the better prepared to administer to the sufferers who may fall into our hands for treatment:

1. Irritation; 2. Stasis; 3. Congestion; 4. Exudation. Up to this point the process is regular, whenever not arrested by sufficient cause.

We may now name as the fifth stage terminations, so called, of this process, without which it can not be said to be complete: 1. Resolution; 2. Organization or induration,—either of which may be attained without destruction of the part, which may immediately return to a state of health in the former, and mediately through this in cases of the latter. But *re*-solution may be effected in two ways—one fatal to the part, the other benign and conservative thereof.

That which is fatal, is the solution of decomposition of the blood crasis, chemically, or by devitalization; and constitutes the worst form of that which has been termed sphucelous or mortification; while the benign may pass either the ordeal of simple re-solution and resorption; or that form of re-solution which is termed suppuration; (which in its most benign form is also resorbed at once or after longer or shorter periods of encystment,) and rupture and discharge of the broken-down exudate with a little cellular tissue involved therein, restoring the parts to normal strength and activity.

Now, these stages are not perceptible to the eye, although so painfully patent to the erudite mental apprehension.

I do not pretend that these stages in name are new, but that which is new to the mass of those who have to do with pathological states is, correct and definite apprehension and use of the terms and the states which they indicate.

Writers and speakers do not limit irritation to a single tissue as its seat; neither do they limit congestion, as it should be, to the vascular system, (although in a sense, nerves may be congested, but by an unseen agent ignored by the mass of those who acknowledge and call themselves pathologists).

Let us ask them, what irritation is? It is disturbance of the "me" in its most occult relationship to organism in the sea of nerve-aura in the mucoid neurine, within and without the perceptible nervous tracts of the living body; this presence in this location alone constitutes the body a sentient point or individual.

If this be apprehended, it is plain, then, that irritation is a dynamic result that may be purely dynamic in its origin or mechanical in its inception,—thus limiting all disease to something more occult than perceptible or material organization, in the ravages of which we alone cognize its presence by the eye. And what are we to understand by the term Stasis? Simply a standing-still of the blood-column within the capillaries of the part in which the mischief is, while the general current continues its regular flow; which continuance is the immediate cause of *congestion.* And now let us ask what congestion is? An undue fullness of the capillaries *behind* the point of stasis; which, if continued, will result in exudation. This last is nothing but a "weeping through" the thinned reticular walls of the capillaries of the more fluid portions of the blood.

There is such a state of relaxation of these fine vessels, as to permit even blood corpuscles to escape into the cellular tissue, producing "pete chiæ" (flea-bites).

A frequent exudate in low forms of nervous fever, is one of dark grumous appearance, consisting of the broken-down or devitalized blood-mass in which the cruorine is diffused, producing the unsightly aspect; this has been the source of much anxiety among the ignorant attendants upon such cases, mistaking it for diffusive mortification.

And indeed it is an untoward symptom, and demands prompt oxygenation of the circulation if the patient is to recover.

It will be seen that we make no place for mortification as distinct termination of the inflammatory process, for the obvious reason that it is but the chemical change through which the material elements of the body or part pass in consequence of the absence of the systemic and molecular life. What is supuration? Simply a less degree of vital *negation* of which sphacelus is the type.

The stages of the inflammatory process, then, may be regarded as nothing short of death in the ratio of the presence of the degrees of the stages enumerated,—the process or the system, or part, yielding to the conquering force; thus putting an end to the contest between the tendency to health on the part of the system, and death and disintegration on that of disease

